# The RNA-Binding Function of Ribosomal Proteins and Ribosome Biogenesis Factors in Human Health and Disease

**DOI:** 10.3390/biomedicines11112969

**Published:** 2023-11-04

**Authors:** Caterina Catalanotto, Christian Barbato, Carlo Cogoni, Dario Benelli

**Affiliations:** 1 Department of Molecular Medicine, Sapienza University of Rome, 00185 Rome, Italy; caterina.catalanotto@uniroma1.it (C.C.); carlo.cogoni@uniroma1.it (C.C.); 2 National Research Council (CNR), Department of Sense Organs DOS, Institute of Biochemistry and Cell Biology (IBBC), Sapienza University of Rome, 00185 Rome, Italy; christian.barbato@cnr.it

**Keywords:** ribosomal origins and evolution, RNA-binding proteins, ribosomal RNA, ribosomopathies, DBA, SBDS

## Abstract

The ribosome is a macromolecular complex composed of RNA and proteins that interact through an integrated and interconnected network to preserve its ancient core activities. In this review, we emphasize the pivotal role played by RNA-binding proteins as a driving force in the evolution of the current form of the ribosome, underscoring their importance in ensuring accurate protein synthesis. This category of proteins includes both ribosomal proteins and ribosome biogenesis factors. Impairment of their RNA-binding activity can also lead to ribosomopathies, which is a group of disorders characterized by defects in ribosome biogenesis that are detrimental to protein synthesis and cellular homeostasis. A comprehensive understanding of these intricate processes is essential for elucidating the mechanisms underlying the resulting diseases and advancing potential therapeutic interventions.

## 1. Introduction

**Figure 1:**
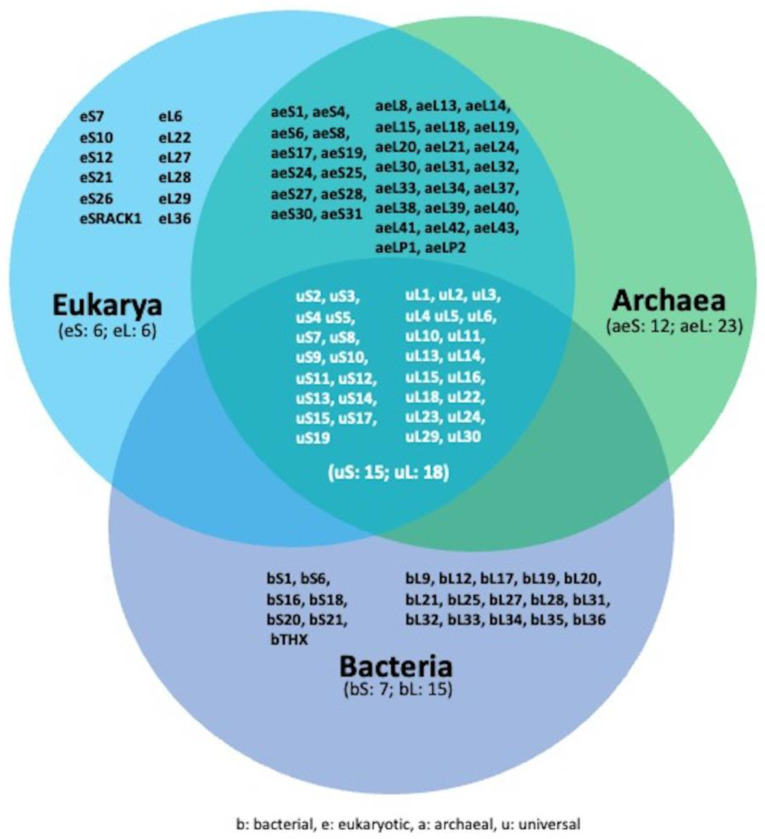
Schematic depiction of the evolutionary distribution of ribosomal proteins across superkingdoms. Universal ribosomal proteins are depicted in white within the central area of the three intersecting circles. Ribosomal proteins specific to bacteria are located within the purple section of the lower circle, while those specific to eukaryotes can be found in the light blue section on the left. Ribosomal proteins exclusive to archaea are situated within the region emerging from the overlapping light blue and green circles. Notably, all archaea-specific ribosomal proteins are also present in eukarya, indicating the absence of any ribosomal proteins unique to archaea. The nomenclature of ribosomal proteins is consistent with the literature criteria [11].

RNA-binding proteins (RBPs) are a broad group of proteins that specifically bind to RNA molecules, forming ribonucleoprotein particles (RNPs) through interactions with their RNA-binding domains (RBDs), including RNA recognition motifs (RRMs), K homology (KH) domains, double-stranded RNA-binding domains (dsRBDs), intrinsically disordered regions (IDRs) and others [1]. RBPs play a crucial role in numerous biological processes of all living organisms, ranging from transcription to splicing, from modification to intracellular trafficking, and from translation to decay. To date, more than 1500 RBPs have been identified in humans [2,3], and their activity and regulation are essential for the proper function of many biological processes. Therefore, it is not surprising that the impaired function of RBPs can lead to the development of a wide range of diseases, ranging from genetic disorders to cancer [4,5,6,7]. Among the plethora of RNPs, ribosomes can be considered their archetype, having appeared in their archaic form about 4 billion years ago and remaining highly conserved throughout extant life (Figure 1) [8,9,10]. Current ribosomes are macromolecules composed of ribosomal RNA (rRNA) and ribosomal proteins (RPs), whose composition is believed to have been selected during evolution to contribute to the improved fitness of RNA molecules with peptidyl transferase activity (PTA) and to ensure accurate and efficient reading of the genetic code. Numerous ribosome biogenesis factors (RBFs), also RBPs, participate in the proper assembly and function of the mature ribosome. Nowadays, ribosomes control the expression of almost all proteins present in living organisms, and the impairment of their functions results in the emergence of a set of diseases, including ribosomopathies and cancer. The objective of this study is to focus attention on the modified RNA-binding activity of RB and RBF as a causative factor leading to impaired ribosomal function in the development of certain ribosomopathies. In this context, we aim to provide an explanation for how the altered function of these proteins affects the translation of specific mRNAs, rather than all translatable ones.

## 2. From the RNA World to the Appearance of RNA-Binding Proteins through Ribozymes with Peptidyl Transferase Activity

**Figure 2:**
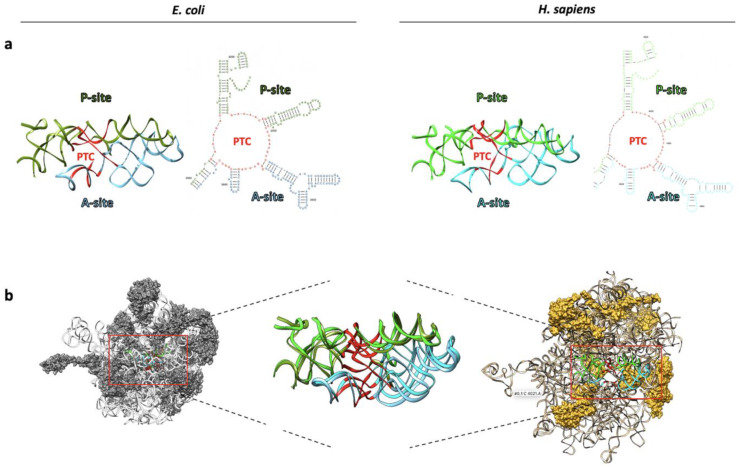
**Functional ribosome cores are universally conserved in all living systems.** (**a**) A three-dimensional representation of the pseudo-symmetry within the peptidyl transferase center (PTC) is depicted in 2D structure and ribbons. The structural representations were extracted from the cryo-EM data obtained for ribosomes from *E. coli* (PDB ID: 7K00) and *H. sapiens* (PDB ID: 4UG0), as depicted on the left and right sides of the figure, respectively. The visualization was generated using UCSF Chimera [21]. Two-dimensional structures of PTC were generated using R2DT software (Version 1.4) [22]. Bases corresponding to the P-site and A-site are denoted by green and light blue letters, respectively, while bases delineating the PTC pore are highlighted in red. This color scheme is consistently maintained throughout the figure. P and A represent two of three active sites of ribosome corresponding to peptidyl and aminoacyl site, respectively. (**b**) In evidence, PTC mapped into the large ribosomal subunit and superimposed.

While contemporary eukaryotic ribosomes consist of approximately equal parts protein and rRNA macromolecules, accumulating evidence over time suggested that rRNA plays a central role in controlling peptide bond catalysis and ensuring the fidelity of genetic code reading. Initially met with skepticism, this perspective has now gained widespread acceptance, leading to the recognition of ribosomes as ribozymes. Indeed, the atomic structures of the large ribosomal subunits in different living species consistently reveal the absence of ribosomal proteins (RPs) at the core of the peptidyl transferase center (PTC) [12,13,14,15]. Similar conclusions were reached in the past by studying the dispensability of different ribosomal proteins while maintaining the integrity of the ribosomal PTC [16,17] and through experiments involving ribosomal inactivation by cleaving a single phosphodiester bond in the rRNAs (Figure 2) [18,19,20].

It has been proposed that the essential functions performed by the RNA component of ribosomes during protein synthesis can be traced back to its ancestral role in the prebiotic environment of primordial Earth. According to the “RNA world” hypothesis, this primordial environment favored the emergence of biological replicators composed of RNA molecules exhibiting behavior similar to genetic algorithms; namely, RNA molecules that could iteratively replicate, mutate, and be selected based on improved fitness resulting from acquired mutations [23,24,25,26]. Some of these mutations enabled certain replicators to fold into structures with enzymatic activity, such as ribozymes capable of peptidyl transferase activity (PTA), ultimately leading to the synthesis of stochastic and non-finalized peptides. Among these peptides, those with RNA-binding activity could have played a pivotal role in the selection of riboswitches with PTA for several reasons:

(1) Stabilizing one of the numerous diverse structures that RNA molecules can fold into, thus slowing down the folding process towards the most functional structure [27,28].
(2) Promoting conformational changes in RNA upon binding to even relatively small peptides, expanding the range of possible RNA structures, as exemplified by “riboswitches” [29].
(3) Preventing RNA misfolding through chaperone-like activity [30,31].

In addition to these reasons, the inherent versatility of proteins, in comparison to RNA molecules, enables them to adopt a wide variety of shapes and three-dimensional structures. This expands their range of functions to the extent that they play a significant role in shaping the skeletons and structures of cells, tissues and organisms. Collectively, the examples described above provide a rationale for the evolution of living systems that led to the emergence of proteins, of which some were capable of binding RNA. Support for this hypothesis is derived from phylogenetic studies demonstrating that ribosomal proteins with RNA-binding activity are among the most ancient and universally conserved proteins [32,33]. It is important to consider that in the prebiotic environment, proto-peptides were not exclusively generated by ribozymes with PTA. In fact, ever since Stanley Miller’s pioneering work in 1953, it has become clear that amino acids coexisted with various other small organic compounds and numerous small peptides in the primordial environment [34,35,36,37,38]. Furthermore, there is evidence of traces of amino acids and simple organic compounds in cosmic dust and meteorites, even in the absence of living organisms [39,40]. However, the remarkable advancement of protein synthesis machinery, as we know it today, goes beyond PTA alone. Instead, it is rooted in the ability to combine PTA with the accurate reading of the genetic code. This has two important implications: first, during evolution, the selection process shifted from nucleic acids to proteins, and second, stochastic mutations that increased an organism’s fitness through the expression of a new protein could be retained in subsequent generations. To better understand the significance of this last feature, let us consider the existence of ribozymes that translate non-coded peptides with zero probability of being identical to each other. In this scenario, the proteins produced by a ribozyme would exhibit subtle differences from one another [23]. Some of these newly synthesized peptides could enhance the fitness of certain RNA replicators. However, for the information contained in their sequences to be preserved in subsequent generations, the system must ensure a certain level of reading fidelity. In other words, the evolutionary success in perpetuating variants with higher fitness is achieved through the selection of proteins that reduce errors during the translation process, as in the case of proteins which, by directly binding rRNA, regulate the fidelity of the genetic code reading. Indeed, both rRNA and RBPs are directly involved in the accuracy of translation by, for example, regulating tRNA binding to the P site [41,42] and binding to rRNA [43,44,45], respectively. In this context, the reduced size of RPs could be attributed to their ancestral origins when translational accuracy was limited. A shorter amino acid chain decreases the likelihood of encoding an incorrect protein, ensuring the success of perpetuating variants with higher fitness. The evolutionary journey that transformed a ribozyme with limited accuracy in reading the genetic code into the present translation machinery, with an error frequency of one error in 10^3^–10^4^ polymerized amino acids, occurred through an extensive series of intermediate stages. Each of these steps was based on the reciprocal interaction between rRNA and RPs. Indeed, the Accretion Model of ribosomal evolution depicts rRNA that recursively accreted and froze over time, increasing in mass [8,46,47]. This model is reinforced by the discovery of temporal correlations (co-evolution) between the acquisition of rRNA elements and RP segments with which they interact [48]. A three-dimensional comparative analysis of ribosomes has revealed sequential acquisitions of capabilities during evolution, including RNA folding, non-coded amino acid condensation to form peptides, subunit association, correlated subunit evolution, decoding and energy transduction [49]. It should be noted that this model is not strictly unidirectional as it is also possible to observe the removal of eukaryotic expansion segments and the loss of RPs following genome reduction, as seen in microsporidia [50]. Expanding the Accretion Model to the evolution of RPs also reveals a hierarchical increase in the complexity of their three-dimensional structures. They progress from simple short random coil (RC) peptides bound to rRNA to proteins elongated and coalesced into secondary structures such as β-sheets and α-helices, [12,17,41,51]. Some are essential for the correct overall folding and assembly of ribosomes, whereas others are not. A few RPs are positioned at or near the subunit interface, where they can influence ribosomal function, while the majority are located on the solvent-exposed surface and are distant from any functional site. Many of these RPs do not exhibit extensive interaction with rRNA, and similarly, it appears that only a few single-stranded nucleotides in rRNA participate in the interaction. Consequently, it seems unlikely that a single, specific interaction could significantly stabilize the RNA tertiary structure. Instead, it is more plausible that strong protein–protein contacts enable the formation of RP complexes that crosslink two or more segments of ribosomal RNA. In summary, we must recognize that the interplay between r-proteins and rRNA is essential for the optimal functioning of the ribosomal machinery. The functional importance of these interactions lies in their cooperative nature. rRNA acts as a molecular scaffold, guiding the precise positioning of ribosomal proteins within the ribosome. These interactions stabilize the ribosomal structure and provide functional surfaces for substrate binding and catalysis during translation. Conversely, ribosomal proteins assist in maintaining the correct folding of rRNA segments, contributing to the overall stability and function of the ribosome.

## 3. Overview of Ribosome and Ribosome Biogenesis

**Figure 3:**
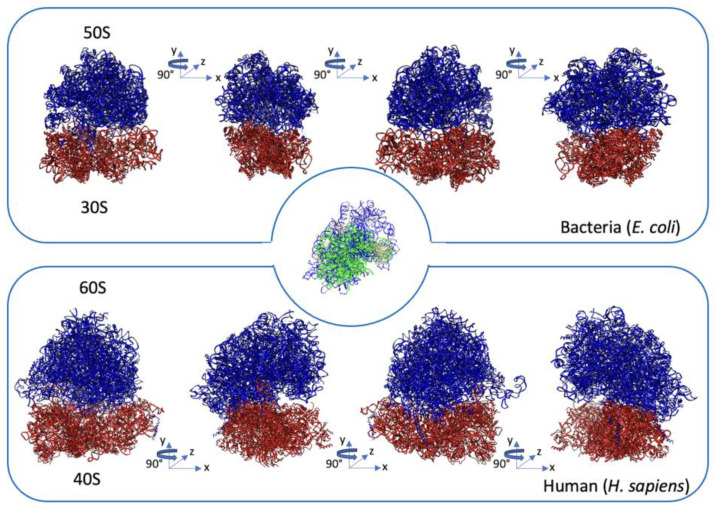
**The structure of ribosomes.** A side-by-side comparison of the structures of prokaryotic (*E. coli*, pdb ID 7K00) and eukaryotic (*H. sapiens*, pdb ID: 6QZP) ribosomes. Ribosomes consist of the large (blue) and the small (red) subunit which comprise a combination of ribosomal RNA (rRNA) molecules and proteins. The rRNA molecules provide the basic building block of the ribosome, establishing its basic structure and functional characteristics. The ribosomal proteins contribute to the overall stability and integrity of the ribosome by bridging structural gaps and promoting the efficient synthesis of proteins [53,54,55]. At the center of the figure, rRNA backbone ribbons extracted from superimposed large ribosomal subunits display noticeable variations in size compared to their conserved three-dimensional conformation. The bacterium (*E. coli*) is green, and the eukaryote (*H. sapiens*) is blue.

The present-day ribosomes consist of both small (30S and 40S) and large (50S and 60S) subunits in prokaryotes and eukaryotes, respectively, and they exhibit a highly conserved three-dimensional structure in all living cells, as shown in Figure 3 [52].

Their reciprocal association forms the 70S complex in prokaryotes and the 80S complex in eukaryotes, creating the integral A (aminoacyl), P (peptidyl) and E (exit) sites. The A site accepts incoming aminoacyl-tRNA, the P site holds the tRNA with the growing peptide chain and the E site accommodates the deacylated tRNA before it departs from the ribosome. The precise order in which amino acids are incorporated into nascent polypeptide chains depends on the accuracy with which the genetic information in the mRNA is encoded by the ribosomes through the coordinate action of the mRNA, tRNA and various translation factors. Achieving this accuracy requires the ribosomes to undergo a complex and energy-intensive process of biogenesis. In eukaryotes, ribosome biogenesis initiates in the nucleolus, which is a specialized nuclear region for ribosome production that involves all three primary RNA polymerases. RNA polymerase I transcribes rDNA to produce the 47S polycistronic precursor pre-rRNA (35S in yeast), which undergoes further processing to yield mature rRNAs: 18S, 5.8S and 28S. RNA polymerase II generates a class of messenger RNAs (mRNA) known as 5′-Terminal-Oligo-Pyrimidine (TOP)-mRNAs, encoding ribosomal proteins (RPs) and ribosome biogenesis factors (RBFs). These motifs help coordinate the regulation of all ribosome biogenesis and translation components [56]. RNA polymerase III synthesizes the 5S rRNA, which becomes part of the large ribosomal subunit [57]. The sequential assembly of RPs and rRNAs relies on a series of transient factors referred to as ribosomal assembly factors (RAFs) or ribosome biogenesis factors (RBFs). These factors include small nucleolar ribonucleoproteins (snoRNPs), nucleases, ATPases, GTPases, RNA helicases and other proteins without predicted enzymatic activity. In eukaryotes, more than 200 of these factors have been identified, and their coordinated interaction is essential for functional ribosome formation [58,59]. Some of these factors are associated with the pre-rRNA 47S to form the 90S pre-ribosome, inducing specific exo- and endonucleolytic cleavages in premature rRNA [60,61]. Others participate in concurrent post-transcriptional modifications of approximately 200 rRNA nucleotides. These modifications include pseudouridylation and 2′-O-ribose methylation. They are catalyzed by two types of small nucleolar ribonucleoprotein complexes: H/ACA box snoRNPs and C/D box snoRNPs, respectively [62,63,64], that together cover around 95% of identified rRNA post-transcriptional modifications, with the remaining 5% involving acetylation or other types of changes [65,66,67,68]. It is worth noting that the nucleotides targeted by snoRNPs are in crucial regions of the ribosome, including the peptidyl transferase and decoding centers. These modifications contribute to both the correct folding of rRNA and, consequently, the proper functioning of ribosomes [69,70,71,72]. Additional ribosome biogenesis factors known as “placeholders” temporarily bind to specific sites on nascent ribosomes until these sites are structurally ready for other factors to take over and prevent premature recruitment of subsequent factors, early formation of structures and potential folding issues [73]. The initial stages of ribosome biogenesis in the nucleolus yield the pre-40S and pre-60S subunits, which are then exported to the cytoplasm for final maturation. The list of factors involved in the correct assembly of functional ribosomes is extensive and the detailed description for all of them is beyond the scope of this review, although their high number gives an idea of the complexity of the process, making it one of the most energy-intensive for cell growth [74]. As a result, rigorous control mechanisms have evolved to ensure the quality of ribosome biogenesis through various cell signaling pathways, including c-Myc, MAPK/ERK and mTORC1. These pathways allow ribosome biogenesis rates to adapt to changing environmental conditions [75,76,77,78]. When normal mammalian cells receive stimuli promoting cell proliferation, they respond by increasing ribosome biogenesis and protein synthesis. The gained ribosome production enables them to meet the increased biosynthetic demands associated with cell division, ensuring that daughter cells possess the necessary cellular machinery for survival and normal function [79,80]. Conversely, exposure to various stressors (such as doxorubicin, replication stress, hypoxia and growth factor deprivation), or the compromised functioning of ribosomes themselves, leads to an immediate arrest of rRNA transcription and subsequent disruption of various steps in ribosome biogenesis. This is accomplished through the activation of nucleolar stress by various routes, involving factors like p53, ARF, PTEN and pRB [81,82,83,84,85,86,87]. In the context of cancer cells, the dysregulation of tumor suppressor genes and proto-oncogenes results in the upregulation of ribosome biogenesis [88]. This, in turn, accelerates cell growth by altering the rate of cell cycle progression. Therefore, changes in ribosome biogenesis rates can be considered a consequence of neoplastic transformation [89]. However, even alterations in protein synthesis levels alone can induce neoplastic transformation. Increased expression of proteins involved in the control of translation initiation, such as eIF4E, leads to changes in mRNA translation, resulting in tumor formation [90,91]. Additionally, evidence from ribosomal disorders suggests that changes in both the quantity and quality of ribosomes can, on their own, shift the pool of translated mRNAs toward promoting neoplastic transformation [92]. However, despite stringent quality controls of ribosome biogenesis, since their first identification, ribosomes appear to be different [93]. The next progressive technical improvements made to their study confirmed the marked diversity of ribosomal particles between different types of cells of the same organism or during the different stages of organism development to such an extent that today we speak of heterogeneous ribosomes [94,95,96]. Sources of diversity arise both from RP content and post-translational modifications (PTMs) of RPs [97] and from rRNA sequences and their post-transcriptional modifications [98,99,100], as well as the type of non-ribosomal proteins bound to them [101] and the substitution of RP paralogs [102,103]. To date there is no univocal and definitive vision regarding the role of the heterogeneous ribosomal architecture and the physiological role for some of their modifications is not yet fully known. However, ribosomes can be perceived as a hub for the integration of a set of spatiotemporal intra- and extracellular signals that would lead to dynamic variations in their composition [104,105,106,107]. At any time, the different combination of PTMs and/or RBPs bound to the ribosomes could change their binding affinity for specific structures or sequence motifs of specific mRNA resulting in alterations of their translational activity [108,109]. Several studies on different types of living organisms have highlighted a functional relationship in this sense [97,103,110,111].

## 4. Ribosomopathies May Be Caused by Impaired RNA-Binding Activity of RPs and RBFs

Based on what is described above, the cooperative interaction between RPs and rRNAs must be considered a prerequisite for the optimal functioning of the RNP complex. Due to the structural complexity of both the molecular machinery and the mechanisms controlling its correct function, it is generally difficult to define a single path for the appearance of the pathological phenotype resulting from their impairment. Indeed, although all ribosomopathies share defects in the production of ribosomes, their appearance can originate from mutations of genes that control different molecular mechanisms ranging from the transcription and modification of pre-ribosomal RNA (pre-rRNA) to its processing until ribosome assembly. As a rule, homozygous mutations of genes coding for RPs and RBFs are lethal, while their single-copy mutations can generate a group of heterogeneous diseases, defined as ribosomopathies, including Diamond-Blackfan anemia (DBA), Shwachman–Diamond syndrome (SDS), X-linked subtype of dyskeratosis congenita (DKCX) and 5q- myelodysplastic syndrome (5q- MDS) [112,113,114,115]. Contrary to what one might expect from dysfunctions of macromolecular complexes present ubiquitously in all cells, the resulting class of diseases shares a series of common and paradoxical characteristics, including hematopoietic defects and skeletal anomalies that relegate the pathological phenotype to specific areas of the body [116]. To date, there is no univocal explanation to justify this phenomenon, although numerous data show that p53 through cell cycle arrest and apoptosis may be the etiological agent of the tissue-specific phenotype of ribosomopathies. As a matter of fact, depletion of p53 has the potential to rescue the clinical manifestations associated with various ribosomopathies [117,118,119]. Other studies instead highlight how mutations in RP or RBF can influence per se the levels of ribosomes and, consequently, also the rate of global protein synthesis [120,121,122,123]. At first glance, it might appear straightforward to assume that the reduced number of ribosomes leads to a global reprogramming of translation, resulting in lower levels of translation for all expressed mRNAs across different cell types. However, several experimental evidence suggest that the concentration of ribosomes plays a crucial role in the translational control of a specific subset of mRNAs, depending on their structural characteristics. Specifically, the 5′ untranslated region (UTR) and its length, as well as the length of the open reading frame (ORF), may determine the loading of mRNAs onto ribosomes [124,125,126]. The strongest evidence is the significant and specific reduction in translational control for those mRNAs expressing proteins involved in erythroid lineage commitment. For some ribosomopathies, detailed studies have revealed the molecular mechanisms that govern the function of proteins derived from genes affected by mutations, highlighting how their defective rRNA-binding capacity is also causative of the reduced ribosomal activity associated with ribosomopathies. Below, we present two emblematic examples of mutated genes expressing RP and RBF associated with DBA and SDS, respectively.

### 4.1. Impairment of Ribosomal Function for Rps19 Protein in Diamond-Blackfan Anemia

DBA corresponds to a hereditary, uncommon form of pure red blood cell aplasia characterized by the incapability of erythropoiesis. The disease condition typically appears within the initial year of one’s life, with 95% of DBA cases diagnosed before 2 years of age [127]. Fifty-five percent of DBA patients have autosomal dominant mutations associated with 1 of 19 genes encoding RPs belonging to small or large ribosomal subunits that cause haploinsufficiency of the corresponding gene expression product [128]. In addition to RPs, several non-RP genes also contribute to the pathogenesis of DBA like *GATA1*, *HSP70* and *TSR2* [129,130]. The frequency of mutations differs among various genes, reaching a maximum value of 25-30%, affecting the *RPS19* gene, which encodes for RP eS19 [131,132]. This protein is a component of the small ribosomal subunit where it interacts with the 18S rRNA through both a series of hydrogen bonds and electrostatic interactions [133]. Both computational and experimental analyses showed that several mutations of the *RPS19* gene reported so far may break some of these hydrogen bonds while other mutations appear to alter the surface electrostatic properties of the protein perturbing interactions between negatively charged rRNA and the positively charged surface of Rps19 [134,135]. Consequently, the inability of mutated RPS19 to interact with 18S rRNA would impair its correct assembly into ribosomes. In addition, Rps19 also participates in the processing of pre-rRNA and in the maturation of the 40S ribosomal subunit, favoring the formation of the decoding site. Its defective function or its depletion causes an impairment of translational fidelity and a reduction in 47S rRNA synthesis [136]. The net result is a reduction in the amount of functional 80S and protein synthesis that induces a competition between the cytosolic mRNAs for the available ribosomes to the detriment of those mRNA essentials for differentiation of erythroblasts. Among these, the GATA1 mRNA, for which the presumed folded structure is assumed by its 5′-UTR, makes it particularly sensitive even to slight variations in protein synthesis [126,137,138]. It has also been hypothesized that the tissue-specific character of ribosomopathies may originate from the existence of specialized subgroups of ribosomes with increased affinity for 5′-UTR of transcripts mostly involved in erythroid differentiation. However, stoichiometric analysis of RPs isolated from ribosomes belonging to four cell models of erythropoiesis showed a consistent protein balance throughout the process of cellular differentiation, thus ruling out in this case the possibility of ribosomal diversity as a source of DBA ribosomopathy [139].

### 4.2. Impairment of Ribosomal Function for SBDS Protein in Shwachman–-Diamond Syndrome

Shwachman–Diamond syndrome (SDS) is a rare autosomal recessive disorder characterized by bone marrow failure, exocrine pancreatic dysfunction and a predisposition to leukemia. The age of onset is heterogeneous, being able to occur in the prenatal, neonatal and infantile periods [140]. Approximately 90% of SDS patients carry mutations in the Shwachman–Bodian–Diamond Syndrome (SBDS) gene, which encodes the SBDS protein of 28,764 Da [141,142]. However, ~10% of patients do not exhibit mutations in the *SBDS* gene. In these cases, other mutated genes such as DnaJ heat shock protein family (Hsp40) member C21 (DNAJC21), elongation factor-like 1 (EFL1) and signal recognition particle 54 (SRP54) are associated with an SDS-like phenotype [143]. The expression products of these genes participate in a common pathway involved in the maturation of the 60S ribosomal subunit. Specifically, SBDS is involved in the late stage of 60S ribosomal subunit maturation in the cytoplasm. It collaborates with the GTPase elongation factor-like 1 (EFL1) to facilitate the release of eIF6 from the large ribosomal subunit [144,145,146,147]. The eIF6 protein binds to the sarcin–ricin loop (SRL) of 28S rRNA, uL14 and eL24 on the intersubunit face of the 60S subunit, inhibiting its association with the 40S particle [148,149]. SBDS occupies multiple sites on the 60S subunit, spanning from the P site to the peptidyl transferase center (PTC), while EFL1 binds to the forming GTPase-associated center (GAC) of the large subunit. The concerted action of SBDS and Efl1 enhances the rotational dynamics of SBDS through its flexible region, which interacts with rRNA to promote the release of eIF6 [150]. The interactions of SBDS and EFL1 with the pre-60S subunit serve as a final quality control system for the integrity of both the P-site and the GAC. The subsequent release of eIF6 from the 60S subunits signifies that the 60S particle is mature and ready for translation. Certain disease-associated variants of SBDS have demonstrated impaired binding to ribosomal RNA, hindering proper folding into dynamic conformational states necessary for initiating the release of eIF6 from the 60S ribosomal subunits [115,150]. The increased retention of eIF6 inhibits the joining between ribosomal subunits, resulting in reduced protein synthesis. In transgenic mice, overexpression of eIF6 mimics the phenotypes of ribosomopathies characterized by defective hematopoiesis, while a series of suppressor mutations targeting eIF6 rescue the disease-associated phenotype [144,151]. Subsequent X-ray crystallography analysis of the binary complex 60S/eIF6 revealed that suppressor mutations affecting amino acids utilized by eIF6 to interact with the surface of the 60S subunit included those amino acids favoring binding with the SRL of 28S rRNA [148,149]. Additionally, for SDS, it has been demonstrated that the reduction in available mature ribosomes impacts translation at the level of re-initiation of mRNAs whose products are involved in the regulation of granulocytic differentiation [151,152,153].

## 5. Conclusions

The ancestral origins of the ribosome and its evolutionarily conserved essential functions make it possible to consider it as an excellent reference model for understanding the evolutionary meaning of RNA–protein interactions that are useful for the emergence of living organisms. Indeed, the primitive heart of the ribosome made up of RNA with ribozyme functions has represented a source for the synthesis of new proteins from which those capable of binding the RNA itself have, in turn, represented a driving force to further improve its enzymatic activity and reading fidelity. Their functions, however, are not attributable to the interaction of a single and specific couple of RNA/proteins but are the result of an intricate interconnected network composed in eukaryotes by 79 ribosomal proteins (RPs) and four ribosomal RNAs (rRNAs). The complexity of a system also results from its ability to keep the different and distant sites that compose it in communication with each other. Remarkably, the system is further complicated in its understanding by the numerous factors participating both in the assembly process and in the surveillance mechanism for the preservation of ribosomal integrity. Understanding the specific contribution of each RNA–protein interaction to the proper functioning of the above represents one of the major challenges in the current ribosome research which can be prosecuted through interdisciplinary and integrated approaches ranging from structural, phylogenetic and mathematical methods. Looking ahead, utilizing emerging methods such as in vivo single-molecule tracking [154] and cryo–electron tomography [155,156] will afford a vital comprehension of the assembly process of ribosomes within their intricate native cellular milieu. Furthermore, the formulation of predictive models elucidating pathogenicity of missense mutations through artificial intelligence [157] coupled with the functioning of complex cellular processes within a living cell will enable better integration and understanding of those exome sequencing data of patients revealing further gene variants associated with ribosomopathies that are negative for mutation of known genes.
